# A comparative review of Haute Autorité de Santé and National Institute for Health and Care Excellence health technology assessments of Ikervis® to treat severe keratitis in adult patients with dry eye disease which has not improved despite treatment with tear substitutes

**DOI:** 10.1080/20016689.2017.1336043

**Published:** 2017-08-03

**Authors:** Yasmina Iffet Eroglu

**Affiliations:** ^a^ Laboratoire de Sante Publique, Faculte de Medecine, Aix-Marseille Universite, Marseille, France

**Keywords:** cationic emulsion, cyclosporine A, ocular delivery, dry eye disease, severe keratitis, ophthalmology, topical, drug compounding, composite end point, reimbursement

## Abstract

**Background:** In 2015, Ikervis® became the only EMA-approved cyclosporine A (CsA) eye-drop for the treatment of severe keratitis in adult patients with dry eye disease, which has not improved despite treatment with tear substitutes. Since the 1980s, CsA has been used empirically for ocular conditions in veterinary medicine then in humans. However, its extremely low aqueous solubility led to its administration in vegetable oils, which is characterized by low ocular availability, poor intraocular penetration, poor tolerability and short shelf-life. Concentrations from 0.05% to 2% are compounded on an industrial scale and reimbursed throughout Europe. In France, Ikervis® has been granted an ASMR score of 5 by HAS, whereas in UK NICE endorsed its use. **Objective:** To review the dry eye disease environment, its challenges and available treatment options, and compare the NICE and HAS assessments to question HAS’ decision to maintain full reimbursement of compounded CsA formulations in the absence of evidence, while reimbursing the EMA-approved drug at 15%. Method: extensive search on PubMED. **Results:** Comparator selection, composite score assessment and use of CE model are key differentiators. **Conclusion:** In topical formulations, improvements to the vehicle are key innovations that can bring significant benefits. After the USA, a Compounding Act is needed in Europe.

## Background on dry eye disease

In studies conducted in the USA and Australia published between 1997 and 2007, the prevalence of dry eye disease (DED) has been assessed to range between 5.5% and 16.6%. Female sex and older age have been found to increase the risk for DED. However, it is thought that some of the variation in observed prevalence between studies derives from differences in the definition of disease used, with the less restrictive definition (i.e. based on only one symptom) driving the higher estimates. Therefore, the true prevalence of moderate to severe DED lies somewhere closer to the lower bound of the range [1].

According to the 2007 International Dry Eye Workshop (DEWS) definition, DED – also known as keratoconjunctivitis sicca (KCS) – is a multifactorial disease of the tears and ocular surface that results in symptoms of discomfort, visual disturbance, and tear-film instability, with potential damage to the ocular surface. It is accompanied by increased osmolarity of the tear film and inflammation of the ocular surface [2]. Reduced tear volume and an increase in inflammatory cytokines are the tear-film alterations seen in DED. Thus, a vicious circle is initiated, where hyperosmolarity, ocular inflammation, and apoptosis may induce damage to the ocular surface, including keratitis [3]. Similar to any chronic inflammatory condition, it will continue to deteriorate if left untreated. Symptoms of DED include discomfort, visual disturbance, and tear-film instability, with potential damage to the ocular surface. Complications associated with DED include conjunctivitis, corneal ulceration, and corneal infection [2].

The two classes of DED are aqueous tear-deficient dry eye and evaporative dry eye, which are not mutually exclusive. Aqueous tear-deficient dry eye may be caused by Sjögren’s syndrome (SS), where the lacrimal and salivary glands are targeted by an autoimmune process. Secondary SS consists of features of primary SS, together with the features of an autoimmune connective disease, such as rheumatoid arthritis or systemic lupus erythematosus among others [2].

A literature search by Wei and Asbell, using the keywords ‘dry eye’ + ‘inflammation’, revealed a total of 458 publications in English between 1 January 1900 and 30 August 2013. These demonstrate that DED is an autoimmune disease of the ocular surface and that inflammation plays a key role in determining its progress and resolution. That inflammation plays the key role in the pathogenesis of dry eye is evidenced by research utilizing tissue culture, animal models, and patients. According to them, the chronicity of the disease suggests that immune mechanisms’ dysregulation leads to a cycle of continued inflammation, accompanied by alterations in both innate and adaptive immune responses. Accordingly, DED has the same core mechanism as other diseases displaying basic characteristics of inflammation, such as atherosclerosis and rheumatoid arthritis [4].

There are seven commonly used tests to measure signs and symptoms of DED: tear osmolarity, Schirmer’s test, tear break-up time using fluorescein, corneal staining, Meibomian grading, conjunctival staining for signs, and the Ocular Surface Disease Index (OSDI) score for symptoms [5]. Such multidimensionality presents both clinical and statistical challenges in terms of efficacy assessment, so it is important first to identify what constitutes clinical benefit for the patients [6].

The challenge of DED is the disconnect between what patients with dry eye complain of and what ophthalmologists can observe and measure [7]. A patient complaining of significant dry eye symptoms may have normal objective test results, while another patient may exhibit clinical signs of dry eye yet feel absolutely comfortable [8]. Physiological mechanisms can partly account for these discrepancies. In early or mild DED, the presence of hyperalgesia can cause significant ocular discomfort without any signs of tissue damage. However, in more severe or chronic disease, decreased corneal sensation can actually reduce discomfort [9].

In a retrospective analysis, among patients who showed evidence of DED by consensus of clinical signs, little more than half of them reported symptoms consistent with a diagnosis of DED [5]. The author pointed to the risks associated with this disconnect – mainly the unlikelihood that these patients would have been given a diagnosis of DED in a clinical setting had the full panel of signs and symptoms not been evaluated. It is important to identify dry eye, even in asymptomatic patients, as it may affect surgery outcomes and contact-lens tolerance [10].

In a cross-sectional association study published in 2017, Vehof et al. demonstrated that chronic pain syndromes (such as irritable bowel syndrome, fibromyalgia, and chronic pelvis pain) are the strongest predictor of a discordance between symptoms and signs. Depression, osteoarthritis, allergy, and atopic disorders were also highly associated with greater DED symptoms to signs, whereas SS and graft-versus-host disease were associated with lesser symptoms to signs. This study showed that lower self-perceived overall health leads to greater symptom reporting than signs would suggest [11].

As symptoms drive patients’ presentation to the ophthalmologist, symptomatic relief is a key clinical benefit of any DED treatment, whereas addressing signs, which may deliver significant benefits over the patients’ disease progression and morbidity, may be overlooked due to the disconnect between symptoms and signs. Thus, it is imperative to treat DED patients with severe keratitis to avoid disease progression and the long-term consequences of inflammation, including ulceration and perforation leading to visual impairment and damage to corneal nerves [2,12], as well as the negative impact on functional visual acuity, resulting in impaired vision, ocular fatigue, and inability to read or drive [9]

Unfortunately, despite data supporting the importance of DED as a public-health issue, the long-term course of the disease, both treated and untreated, is still not well characterized [1,13]. A survey of patients with an average duration of DED of 10.5 years, recruited from two large longitudinal studies of health-care professionals (HCP) in the USA, demonstrated that patients who reported more severe symptoms in the past were more likely to experience worsening over time and to have corneal staining, thereby identifying one clinically relevant indicator of probability of disease progression [1,13].

The human burden of disease remains quite significant compared to other medical conditions, as it has been reported that patients without co-morbidities expecting to live 10 more years would give up 1.6 years of that time to be rid of severe DED, which puts the disease on the same burden level as moderate to severe (class III–IV) angina [14].

In a cross-sectional study to evaluate economic and quality-of-life impact of SS in women, it was demonstrated that DED symptoms affected approximately 60% of patients and interfered with effectiveness at work in nearly 38% of patients [15]. Indeed, DED has a substantial economic burden, where indirect costs make up the largest proportion of the overall cost due to a substantial loss of work productivity. The disease also has a substantial negative impact on physical and psychological function and health-related quality of life [16]. In terms of cost to society, Clegg et al. demonstrated that DED does not impose a direct burden to the health-care expenditure in the European Union (EU). However, the true societal costs are higher, as many dry eye sufferers self-treat with over-the-counter (OTC) artificial tears [17]. According to a survey done in 2171 patients by Yu et al., DED poses a substantial economic burden on the payer and society, as in terms of mean work days lost per patient due to affected performance, severe DED patients lose >128 days per year compared to 91 and close to 95 for mild and moderate patients, respectively [18].

## Current treatment of DED

The current DED therapies meet seven treatment strategies: (1) tear supplementation through use of artificial tears/lubricants; (2) tear retention through use of devices for punctual occlusion, moisture chamber spectacles, and contact lenses; (3) tear stimulation through use of secretagogues; (4) biological tear substitutes such as serum or salivary gland autotransplantation; (5) anti-inflammatory therapies such as cyclosporine A (CsA), corticosteroids, and tetracyclines; (6) essential fatty acids; and (7) environmental strategies through avoidance of triggers [19].

Symptoms are driving patient presentation to the HCP. Therefore the mainstay treatment of DED is symptomatic in the form of tear replacement with so-called artificial tears, which are hypotonic or isotonic buffered solutions that are sold OTC. The denomination ‘artificial tears’ is a misnomer, as most do not mimic the composition of human tears, but rather function as ocular surface lubricants. The lubrication obtained depends on their composition, physical properties, and mechanism of action [20]. However, a lack of symptoms is not a reason for withholding treatment, as patients may have signs (ocular surface staining, early tear break-up, tear hyperosmolarity) in the absence of symptoms [10].

A better understanding of the pathophysiology of dry eye resulted in the realization that simply hydrating and lubricating the ocular surface is inadequate. Inflammation, tear composition and dynamics, and preservation of the delicate homeostasis of the ocular surface are key considerations in treating dry eye. While it is not yet possible to eliminate symptoms completely, it is possible to improve the patient’s condition significantly [8].

According to DEWS, patients for whom first-level therapy (avoidance of external triggers, artificial tears) is inadequate should move to second-level therapy, including anti-inflammatories, secretagogues, and moisture chamber spectacles. In terms of anti-inflammatory treatment options, only some CsA and corticosteroid formulations could demonstrate level 1 evidence (evidence obtained from at least one well-designed, randomized controlled trial [RCT] or evidence from well-designed studies applying rigorous statistical approaches), and tetracyclines are used in occurrences such as acne rosacea and meibomianitis. Secretagogues are not available in Europe. The level of evidence supporting the efficacy of moisture chamber spectacles is quite limited [19].

A systematic review of RCTs on topical CsA for the treatment of DED published in 2014 highlighted several trials with up to 48 weeks of CsA use [21]. In 2005, an 0.1% CsA eye-drop formulation was used twice daily for up to 3 years by patients with moderate to severe DED in a Phase III setting, and it was found to be safe and well tolerated [22]. However, long-term use of corticosteroid eye drops is associated with an increased risk of side effects such as intraocular hypertension, ocular infections, and cataract [23].

T cells appear to play a significant role in the pathogenesis of DED. Studies have shown that CsA can address the cause rather than being merely palliative as artificial tears are. It has been demonstrated that CsA reduces conjunctival interleukin-6 levels, decreases activated lymphocytes in conjunctiva, reduces conjunctival inflammatory and apoptotic markers, and increases conjunctival goblet cell numbers [8]. Increased lacrimation, even in the absence of a deficit in tear production, was demonstrated as a serendipitous effect of CsA as early as 1990 [24,25].

## Thirty years of cyclosporine use in DED

A search on PubMED using the keywords ‘cyclosporine’ and ‘dry eye’ yielded 358 results [26]. The first trial describing (oral) CsA use in DED in SS patients was published in 1986 [27]. Subsequent research focused on the canine population, as DED is the major cause of chronic or recurrent conjunctivitis in dogs, and inappropriate or insufficient treatment due to delayed or wrong diagnosis leads to progressive corneal scarring and blindness [28]. Accordingly, topical CsA has been used by veterinary ophthalmologists since 1989 for the treatment of ocular surface inflammatory disease and KCS in dogs, cats, and horses, and an ointment preparation of 0.2% CsA, Optimmune® (Schering-Plough), was approved by the Food and Drug Administration (FDA) for use in dogs in the management of chronic KCS in August 1995 and chronic superficial keratitis in August 1997.

In his 1997 review on topical CsA therapy, Williams concluded ‘given that topical CsA has been so widely accepted as a valuable ophthalmic preparation in the veterinary world and licensed as such, it is surprising that human ophthalmologists have not employed the drug to the same extent for conditions from vernal keratoconjunctivitis to Sjögren’s syndrome’ [29].

In 1993, a six-week trial in 25 DED patients using a CsA 1% ophthalmic ointment versus placebo [30] and, in 1994, a well-controlled trial with a topical CsA 2% olive oil formulation versus placebo were conducted in 15 DED patients with secondary SS [31]. As a result of these conclusive trials, CsA formulations ranging from 0.05% to 2% ophthalmic emulsions in olive or castor oil have been used in clinical practice up to four times daily as an alternative to corticosteroids in severe forms of DED for several decades [32], compounded from Sandimmune® injectable, solution, or oral tablets. There is a multiplicity of formulations available, but none are listed in the European Pharmacopoeia [33,34].

A 0.05% CsA anionic ophthalmic emulsion was then evaluated in large multi-centre randomized double-masked FDA clinical trials, resulting in Restasis® (Allergan, Inc.) gaining FDA approval in 2003 for twice daily (bid) use in patients whose tear production is presumed to be suppressed due to ocular inflammation associated with KCS. Initial submission was rejected in 1999 by the FDA, as the expert panel voted unanimously against its approval [35]. At the time of Ikervis® (Santen S.A.S.) registration, Restasis® (0.05% CsA; Allergan) was available in some EU countries under compassionate-use programmes. In other countries, compounded oily CsA formulations were used [32,36].

Thus, in 2013, prior to the launch of Ikervis®, three types of CsA were used and 100% reimbursed across Europe, although none were approved by the European Medicines Agency (EMA): (1) Restasis® (0.05% CsA; Allergan) was authorized for compassionate use on a named-patient basis in both France and the UK, as well as in other European countries; (2) Optimmune, a 0.2% CsA (MSD Animal Health) veterinary ophthalmic ointment, was used in several European countries, including the UK but not France [36]; and (3) hospital-compounded CsA formulations were available in many European countries, including France and the UK, whereas pharmacy-compounded CsA was available in the UK but not France [36]. In the UK, the main provider was Moorfields Pharmaceuticals of 0.05% and 2% eye-drop formulations. In France, there were eight main public hospitals providing CsA formulations in concentrations ranging from 0.1% to 2%. In terms of use, one to four drops per day in both countries have been reported as standard practice [36].

Due to the various concentrations and daily dosages used, as well as the fact that the price of compounded formulations is not publicly displayed, it is quite difficult to come up with an average price of compounded CsA in the DED indication. Also, data on the dosage used in the severe keratitis patient subset are not available.

CsA as an active ingredient is difficult to compound, as it has very low solubility in water and water-based solutions. For this reason, many compounded formulations contain alcohol to facilitate the dissolution of CsA [34]. Chast et al. described the issues encountered due to the excessive amount of alcohol in compounded CsA formulations. Most importantly, alcoholic substances have important toxicity properties due to the pro-inflammatory activity of alcohol. This effect is compounded by the specificities of the disease: the patients have severe local symptoms, often painful. At the commonly used concentrations of 1–2% of CsA eye drops, the formulations contain a final ethanol concentration of 6.6–13.2%, which is usually toxic in animals and humans [34]. Also, the conservation of this type of preparation is poor, that is, 1% CsA eye drops prepared from injectable CsA and artificial tears are stable for 28 days in the fridge and 7 days at room temperature [37]. An alternative is to use oral instead of injectable CsA in compounding. The resulting eye drops contain 2.5% ethanol, which is well tolerated locally [34]. Market research commissioned by the manufacturer to GfK in March 2013 highlighted that two-thirds of compounded CsA in France is done based on injectable CsA and the remaining third originating from oral CsA [38].

Therefore, over the last 20 years, various eye-drop formulations of CsA have been used off label in most European countries and without evidence for efficacy and safety in well-controlled RCTs for a variety of therapeutic indications: DED, atopic keratoconjunctivitis, vernal keratoconjunctivitis, corneal graft rejection and graft-versus-host disease, autoimmune diseases such as SS and Stevens–Johnson syndrome, as well as other inflammatory diseases such as corneal ulcers [36]. This widespread off-label use of topical CsA demonstrates the existence of a significant unmet need for effective and well-tolerated options for the treatment of inflammatory or autoimmune diseases in the field of ophthalmology.

In DED, the main challenge is for the clinical targets to meet both signs and symptoms. Restasis® (0.05% CsA’; Allergan) failed to meet end points on signs and obtained an indication in tear production in the USA [39]. Ikervis® failed to meet the composite end point due to symptoms in one of the studies and obtained an indication on signs of dry eye (keratitis) in Europe [40]. Both options benefit from evidence in RCTs as well as real-world evidence, which represent a significant advance over the various unlicensed CsA formulations.

## Ikervis®

Ikervis®, a 0.1% CsA cationic emulsion, obtained EMA approval in April 2015 in the treatment of severe keratitis in adult patients with DED which has not improved despite treatment with tear substitutes [40]. A dossier was submitted for reimbursement in the UK and France. In Germany, the G-BA accepted the health technology assessment (HTA) waiver. Therefore, Ikervis® was not appraised. The decision was driven by the loss of the data protection of CsA.

The clinical trials program for IKERVIS® consists of four studies: two Phase II and two Phase III. The application was primarily based on the pivotal Phase III SANSIKA study, a randomized, double-masked, vehicle-controlled multi-centre European study that assessed IKERVIS® for the treatment of DED in patients with severe keratitis which did not improve despite treatment with tear substitutes. Data from the supportive Phase III SICCANOVE study in moderate to severe DED patients was also provided. The choice of the SANSIKA target population was based on *post hoc* SICCANOVE study results, which suggested a pronounced effect of IKERVIS® in the most severely affected patients (i.e. Corneal Fluorescein Staining [CFS] = 4 and OSDI ≥23). Furthermore, the outcomes of a meta-analysis of the Phase III studies were also provided, including SANSIKA and only the severely affected patients in SICCANOVE [32]. These data were provided to both the EMA and the health assessment technology bodies.

In the SANSIKA study, the proportion of patients achieving an improvement of ≥2 grades in CFS and a 30% improvement in symptoms (OSDI) by month 6 was 28.6% with IKERVIS® versus 23.1% with vehicle (*p* = 0.326) as a primary end point. As secondary end points, Ikervis® showed significant efficacy on the CFS score (*p* = 0.037), as well as a significant reduction in ocular surface inflammation, as assessed by HLA-DR expression (*p* = 0.021), thereby supporting the efficacy of Ikervis® and the ability of its formulation to deliver CsA at the site of action [41].

However, the depth of response at 6 months has been demonstrated *post hoc* to be statistically significant, as the proportion of patients achieving an improvement of ≥ 3 grades in CFS and at least a 30% improvement in symptoms (OSDI) by month 6 was 18.8% with IKERVIS® versus 7.8% with vehicle (*p* = 0.016) [41].

In France only, an additional six months of real-life data was provided from the Temporary Authorisation for Use that had started in October 2013 for 104/890 patients who received the drug. Five per cent of patients had no keratitis left, while 57% showed marked improvement in keratitis and 38% stabilization [42]. These data were not taken into consideration for the assessment.

The outcome of the National Institute for Health and Care Excellence (NICE) evaluation was that CsA (Ikervis®) ‘was a cost-effective use of [National Health Service] NHS resources for people with severe keratitis in adult patients with DED, which has not improved despite treatment with artificial tears’ [43].

The outcome of the Haute Autorité de Santé Transparency Committee was that ‘ despite an important therapeutic need and given the lack of clinical data methodologically acceptable, and the uncertainties around the tolerability, especially due to the presence of CKC in the eye drops, the medical service provided by Ikervis® 1 mg/mL is low in the treatment of severe keratitis in adult patients with dry eye that has not improved despite treatment with artificial tears', thereby granting it an SMR [service medical rendu] of 4 (=weak actual medical benefit). Therefore Ikervis® is included in the list of reimbursable specialties, but with an ASMR [amelioration du service medical rendu] of 5 (=no improvement in actual medical benefit) leading to a reimbursement rate of 15% [42].

## Discussion

The clinical assessments of Ikervis® by NICE and by HAS were quite similar. However, interestingly, they led to totally different outcomes due to the fact that the two key topics – namely, the absence of an EMA-approved comparator and the pivotal Phase III trial’s failure to reach the primary end point – were evaluated totally differently. Both impacted the assessment of a third key topic: the innovation brought by the drug.

### Current medical practice and the selection of the comparator

Both NICE and HAS noted that there was no other licensed treatment available. Current disease management practice can involve the use of topical corticosteroids or compounded CsA eye drops. Whereas HAS concluded that there are no clinically relevant comparators in the given indication, for NICE, the Evidence Review Group (ERG) considered that the relevant comparator for CsA was actually the other unlicensed CsA formulations currently used in clinical practice. However, they noted that a robust indirect comparison was not possible due to the absence of trials comparing these CsA formulations, combined with the lack of a common comparator and the differences in vehicles used in each formulation. Therefore, the only valid economic comparison is a cost-minimization analysis assuming that all CsA-based treatments have equivalent efficacy, similar adverse effects, administration, prescribing, and monitoring costs. This was carried out, demonstrating that Ikervis® is less costly on a monthly basis than Restasis® (0.05% CsA; Allergan) but more costly than the other two CsA formulations (ointment and eye drops) currently used in clinical practice in the NHS [43].

Therefore, one critical difference is that whereas NICE accepted the current clinical practice as a standard for comparison, HAS did not. According to Massetti et al., all potentially relevant interventions for the assessed indication should be compared, irrespective of whether they have a marketing authorization in the indication, and HAS recommends comparing the new product to current best practice and routine treatment [44].

‘In the absence of an appropriate active comparator, the applicant used Ikervis® vehicle as a comparator in all studies’ [32]. Indeed, the most appropriate active comparator would have been compounded CsA eye drops or Restasis® (0.05% CsA; Allergan). However, Restasis® (0.05% CsA; Allergan) has not been approved by the EMA, and there is a plethora of CsA eye drops of various concentrations and formulations used throughout Europe, thereby making it impossible to select one appropriate active comparator for the Phase III trial. There are no international or national guidelines regarding the preparation of compounded CsA eye drops and hence no standardization, as each formulation has different properties.

Therefore, the important point to consider is what the definition is of a clinically relevant comparator. As per the 2008 NICE guidelines, a comparator is any therapy routinely used in the NHS, including technologies regarded as current best practice (including no intervention). In France, the Transparency Commission must identify all products of the reference therapeutic class, as well as other products with similar therapeutic objectives and, where possible, identify the most prescribed competitor, the last included in the positive list, and the product with the cheapest treatment cost. In practice, however, therapeutic improvement (AMSR) is not assessed against all medicines or other therapies listed but against the best available and reimbursed treatment [45].

In commercial terms, a comparator should be any treatment alternative that is currently used in the treatment of the specified target patient population, including observation/no intervention. This comparator represents the current standard of care or current clinical practice regardless of license status, and should therefore be the relevant comparator. Indeed, not identifying a clinically relevant comparator leads to the conclusion that the given patient population receives no treatment, which is rarely the case in medical practice. For this reason, it is important to recognize the presence of empirical, off-label treatment. Even though not on label, many drugs are used in clinical practice and are therefore a standard of care, such as corticosteroids in a wide range of inflammatory diseases.

In France, eight hospital pharmacies have compounded CsA in an eye-drop formulation between 2008 and 2010. CsA eye drops, in 0.05%, 0.1%, 0.5%, 1%, and 2% concentrations, are part of the top 45 most compounded products in the biggest number of hospital pharmacies. Indeed, production has been steadily increasing from <35,000 units between 2004 and 2006 to close to 170,000 units between 2008 and 2010 [46]. In the UK, Moorfields Pharmaceuticals (a division of the Moorfields Eye Hospital) manufactures 0.06% and 2% CsA eye drops containing alcohol and maize oil, and imports a 0.02% CsA veterinary ointment from the USA (Optimmune®; MSD Animal Health).

Kauss Hornecker et al. conducted a retrospective analysis on the use of CsA compounded at the University Hospitals Paris Centre over 5 years. They noted a continuous increase in use for all doses (0.05%, 0.5%, and 2%). Close to 6,000 patients were treated in 2013 alone, and they concluded that the prescription of diverse concentrations of CsA eye drops was current practice in both surgical and medical indications, including dry eye patients. Interestingly, the authors also added that Ikervis® registration should have an impact on the use of compounded CsA in DED, which use should henceforth be justified against the labelled alternative, and in which case the prescriber should inform the patient [47].

Therefore, in the presence of such large-scale, industrial-sized production of compounded CsA, it is quite unexpected that HAS does not acknowledge it as a clinically relevant competitor. In this case, there were >80,000 units of compounded CsA produced as ‘stock preparations’ in hospital pharmacies in France in 2009–2010 [46]. Production on this scale is out of scope for compounded pharmaceutical preparations, as it is industrial scale.

In the USA, guidance on compounding commercially available drugs states that prescribers can order them, as long as there is a clinical difference for the patients. Section 503A of the Federal Food, Drug, and Cosmetic Act provides exemptions from new drug approval, labelling with adequate directions for use, and CGMP requirements, so that drugs can be compounded as customized therapies for identified individual patients whose medical needs cannot be met by commercially available products. Examples are allergic patients who cannot tolerate an excipient, elderly patients who cannot swallow a tablet and therefore need a liquid formulation, or children who need a lower strength compared to the FDA-approved drug. The restrictions on making drugs that are essentially copies ensure that pharmacists or physicians do not compound drugs for patients who could use a commercially available FDA-approved product [48].

No similar guidance has been defined by the EMA. In their 2014 paper, Minghetti et al. recognize that compounding has a traditional history linked to the national territory. However, they argue that it is time for a modernization of the regulation of compounding because it is no longer acceptable to have different quality standards among European countries, as the welfare of patients is a common good [49].

There are significant differences between compounded and approved drugs. One can cite, on top of the clinical testing for safety and efficacy which has already been mentioned, the lack of bioequivalence testing, labelling and instructions to patients, adverse event reporting, the type and extent of quality control testing required (i.e. the lack of retesting incoming bulk ingredients), batch consistency, shelf-life, and microbial contamination [49, 50]. According to a retrospective analysis of all FDA-issued recalls for drugs and biological products issued from 20 June 2012 to 31 December 2014, compounding was associated more frequently with contamination than licensed drugs [51]. In both France and the UK, compounded CsA formulations are available in glass multi-dose bottles of 10 mL. According to the DEWS, the single most critical advance in the treatment of DED came with the elimination of preservatives from OTC lubricants. Indeed, the absence of preservatives is of critical importance, as the ocular surface inflammation associated with dry eye is exacerbated by preservatives. However, because of the risk of contamination of multi-dose products, most contain a preservative [19,33].

### Assessment of the primary end-point outcomes in the presence of a composite score

The primary end point in SANSIKA – namely, the CFS–OSDI response – is a composite score of individual measures for signs (CFS) and symptoms (OSDI).

According to a panel of European experts, the combined use of CFS and a symptom-based assessment could provide a reliable ‘front-line’ diagnostic approach for the evaluation of DED severity in those patients whose signs and symptoms of disease associate well. Thus, they recommended that these criteria should be adopted at step 1 of the diagnostic algorithm. However, they also stated that in cases of discordance, further evaluations are needed in order to improve diagnostic specificity [9].

The composite score was selected in order to be able to demonstrate positive outcomes on both signs and symptoms in DED for EMA approval. However, both CFS and OSDI separately were also retained as secondary end points to demonstrate efficacy in each dimension of the composite score, alongside with the HLA-DR immunological biomarker.

In the UK, clinical experts informed NICE that there is no established and standardized measure of response in severe DED and that several measures of signs and symptoms are used in clinical practice in the NHS, including both CFS and OSDI [41]. CFS and OSDI are recognized and validated outcomes used to measure signs and symptoms, respectively, but NICE was concerned that the validity of the composite end point is unknown. The ERG stated that it is unclear whether the CFS-OSDI response is a clinically relevant end point and what the response thresholds should be to define a response. It also noted that the response thresholds would depend on the criteria used for defining severe DED.

HAS noted that the hypothesis that the composite score would require longer-term trials is to be taken into account as the impact on symptoms would be delayed in relation to the impact observed on the corneal lesions. However, it also noted that any outcomes deriving from other than the primary end point should be considered exploratory in view of the failure to reach the primary end point.

According to a recent research paper from Huque et al., using a composite end point as a primary end point that combines clinically relevant individual primary end points that are likely to have small effects or have low frequency of occurrence is a possible approach to ensure that the RCTs are of feasible size in some diseases. However, for composite end points to be useful, there should be some consistency of treatment effects across the components of the composite, or the components should jointly enhance the treatment effect. Indeed, when not all of the components have the same degree of clinical importance, difficulties can arise in interpreting study findings based on composite end points, that is, some of the components may not characterize clinically meaningful treatment benefit either individually or collectively when they are of secondary importance [52].

The challenge in designing clinical trials for dry eye is the lack of correlation between signs and symptoms, and severity. This makes symptomatology alone a poor indicator of severity in some patients, and also a confounding variable in clinical trials [9]. It may also have implications for monitoring the response to treatment both in the clinic and in clinical trials [53]. It has been postulated by Sullivan et al. that each type of measurement provides distinct information about the condition of the ocular surface. It has been demonstrated that normal subjects are often not distinguishable from moderately severe patients just a few weeks later due to the exceedingly high variability from baseline shown using symptom questionnaires. Furthermore, a retrospective analysis of 344 patients across 11 sites in Europe and the USA demonstrated that symptoms alone are insufficient for the diagnosis and management of DED, calling for a consensus of clinical signs that better reflect all aspects of the disease and discourage reliance on symptoms alone [5]. Therefore, the two components of the composite score cannot be deemed equal.

This was recognized by NICE, who looked into the secondary end points, CFS and OSDI measures, separately. However, HAS dismissed the reliability of looking at secondary end points, even though they are the individual score components of the composite score selected as the primary end point.

According to Davis, while it has been argued that secondary end points may not be measured as well as the primary end point, it is not clear why the results of the secondary variable should be conditioned to the outcome of the primary variable. Indeed, if the measurement technique used is the same for each of the treatment groups, randomization insures that the comparison is unbiased. There is thus no reason to ignore the results in a properly randomized trial [54].

The vehicle effect in both Phase III trials has been raised by both NICE, as the ERG considered that improvements seen in the comparator group may be due to the vehicle itself, concomitant use of artificial tears, or both, and by HAS – a non-negligible therapeutic effect has been observed which can be explained by the properties of the emulsion, optimizing cornea hydration.

In topical preparations, the vehicle is an active part of the formulation – hence not a placebo – and is a key differentiator for the brand. The concomitant use of CsA (Restasis®, 0.05% CsA; Allergan) with artificial tears has also been analysed by Sall et al., who evaluated the efficacy of three branded artificial tears in relieving the signs and symptoms of DED when used as a supportive therapy to a CsA-based anionic ophthalmic emulsion in a 6-month randomized, investigator masked, parallel study. In the first two groups, CsA was combined with a preservative-free carboxymethylcellulose 0.5% agent (Refresh Plus®; Allergan) and with a hydroxypropylguar gellable lubricant eye drop (Systane®, Alcon Laboratories, Inc.). In the third group, Systane® was used alone. The choice of artificial tears had significant effects on outcome measures: the combination Restasis®–Systane® was statistically significantly better in reducing the signs and symptoms of DED than Restasis®–RefreshPlus®. Very interestingly, Systane used alone was statistically significantly better than Restasis®–Refresh® for three ocular symptom frequency scales (burning, dryness, and scratchiness) and six Likert acceptability questions [55].

Obviously, the failure to reach the composite primary end point could be directly linked to the fact that the vehicle used in the trial is not a placebo. Also, lubricants (artificial tears) have demonstrated the ability to provide symptomatic relief to DED patients. In the SANSIKA clinical trial protocol, both arms were allowed to use artificial tears.

### Assessment of innovation

The objective of any health-care technology assessment is to determine the value of new treatment alternatives and its incremental value over currently available treatment alternatives, hence the level of innovation it is bringing to the market.

In France, the provided medical value (SMR) which is the ‘basis of the evaluation for the HAS, is a criteria that takes into account the severity of the pathology for which the drug is indicated, and the drug’s data itself, in the given indication, such as the efficacy and side effects, the place in the therapeutic strategy, especially in view of the available alternatives, as well as its interest in terms of public health’ [56]. Hence, only the innovative health-care technologies which can impact the budget of the statutory public-health insurance are evaluated. Therefore, the number of interventions are limited to the ones with significant added medical value (ASMR). These are the interventions for which an ASMR of I-II-III (major, important, moderate) is claimed by the manufacturer in the submission dossier, and which are expected to generate more than 20 mio Euros in annual sales revenue during the second full year of sales [57]. The manufacturer claimed an ASMR of IV to reflect the innovation brought by an efficient reformulation of an active ingredient used empirically over >20 years in various compounded formulations, without evidence and without label. For this reason, HAS did not carry out a health economic assessment, whereas NICE did.

Looking at the ASMR ratings provided through March 2012, <19% of drugs got an ASMR of I-II-III, whereas close to 70% got an ASMR of 5 [57]. As per definition, an ASMR rating of 1 should reflect the innovation brought by the drug, including a proven reduction in mortality. Demonstrating a reduction in mortality via a RCT may take years for chronic diseases, with the exception of certain types of cancer. As a result, many real innovations are excluded from this category.

According to Lallemand et al., an ideal formulation increases CsA residence time in the eye, is well tolerated and easy to administer, avoids systemic absorption, has a long shelf life, and is easy to manufacture [58].

Pharmacokinetic studies have demonstrated that a cationic emulsion is almost twice as better at delivering CsA to the ocular tissues than an anionic formulation. Indeed, to create an electrostatic interaction with the negatively charged cells of the ocular surface, the vehicle should be positively charged (cationic). Ikervis® (cationic emulsion at 0.1% CsA; Santen) has been compared to Restasis® (anionic emulsion at 0.05% CsA; Allergan) in pharmacokinetic studies designed to evaluate the ocular and systemic distribution of CsA following single and multiple dosing. These confirmed that the cationic charge is much more effective in delivering CsA to the cornea than the anionic one (Cmax: 1372 vs. 748 ng/g; AUC: 26,477 vs. 14,210 ng/g), without systemic absorption [59]. This constitutes real innovation and progress in formulating CsA.

The concept of innovation in the pharmaceutical industry is linked to the brand being a New Molecular Entity (NME). However, it is important to determine how innovative it is, that is, does it bring a new approach to treatment, a new mode of action, or is it a new therapeutic class, or is it simply an alternative within an existing therapeutic class (also known as me-too products). The high use of topical formulations set ophthalmology and dermatology apart in the field of medicine. Indeed, not only the drug, but also the vehicle have a key impact on the brand efficacy. Improvements brought to the vehicle are key innovations enabling a better delivery of the drug to the target site, therefore achieving higher efficacy, fewer side effects, less/no systemic distribution, ease of administration, potential for lower dose formulation due to better penetration, better residence time, longer shelf life, and so on, that is, it has been demonstrated that the aqueous solution of CsA shows more toxicity than other formulations, and that the type of oil and the ethanol concentration used influence cell viability [60]. Similar challenges are seen in the respiratory field, where the device enabling the administration of the corticosteroid, beta-blocker, or other active plays a key role in shaping the brand’s efficacy by improving its delivery to the target site of action.

To summarize, the value of innovation in enhancing delivery of actives in topical formulations may be similar or higher to the patients’ well-being than the value of a NME within an existing mode of action class.

## Conclusion

NICE took a current standard practice–driven approach, recognizing current clinical practice and therefore acknowledging the presence of fully reimbursed, off-label, not standardized alternatives that never demonstrated efficacy in a RCT, and postulating the assumption that in the absence of evidence, all these formulations of CsA were deemed equal, thereby granting Ikervis® similar reimbursement conditions. NICE also requested a CE model to validate Ikervis® price versus the compounded CsA formulations, Optimmune® and Restasis®. On the other hand, HAS decided to include only on-label drugs as comparators, thereby disregarding current clinical practice and the presence of an industrial-scale production of diverse off-label compounded CsA formulations, for which efficacy has never been established in RCTs, thereby granting Ikervis® reimbursement conditions significantly inferior (15%) to the ones of the compounded CsA formulations (100%) and Restasis® (100%) – the first ones lack evidence, and the latter one failed to obtain EMA approval.

Interestingly, despite mounting evidence to the contrary [18], HAS postulated that the management of DED is not a public-health priority. It recognizes the unmet need for therapies and bemoans the paucity of epidemiological data available on the severe keratitis patient segment. HAS stated it does not anticipate a major budget impact. Unlike the UK, France has a system in place to track the production of compounded drugs at the national level. Therefore, the budget impact is not an unknown. However, as a CE model is only required for ASMR I-II-III, the fact that Ikervis® did not fit into any of these categories did not enable the use of a CE model in the decision-making process. Comparing current standard practice (compounded CsA formulations) and Ikervis® would have been the more pragmatic approach by enabling the same reimbursement level (100%) through recognition of the value of the EMA approval (product profile validated through RCT, pharmacovigilance, quality, standardized production, etc.) and through using a CE model for establishing a price recognizing the value of innovation versus the price of currently available alternatives. One might argue that only Restasis® (0.05% CsA; Allergan) should be taken as a comparator, as its efficacy and safety profile have been demonstrated in RCTs and subsequently approved by the FDA, which puts it on a more comparable level to IKERVIS® and its EMA approval. Indeed, assuming similar efficacy and tolerability between compounded and licensed CsA formulations is inherently fraught with errors, as the critical role played by the vehicle in topical formulations delivery cannot be discarded.

This case highlights the need to look into innovation in the pharmaceutical industry with a novel eye, and also the need to re-evaluate the rationale for compounding drugs in the 21st century.

As the world population ages, further strain will be put on the resources available to maintain a sustainable health-care system. Therefore, it will be important to pay for demonstrated value. There is a wide range of compounded drugs still used in clinical practice that are fully reimbursed as part of hospital practice, however, that never demonstrated efficacy and safety in RCTs. Upholding compounding in the presence of on-label alternatives is also detrimental to fostering research and development to improve formulations, the more so in surface diseases seen in dermatology and ophthalmology, where securing the active ingredient’s optimal delivery to the site of action depends solely on the vehicle’s properties. In these areas, it is the vehicle which has the potential to deliver additional benefits to the patient through formulation optimization.

Indeed, a similar example was seen in the paediatric field, which was widely overlooked until the EMA brought in new legislation in January 2007, with the objective of improving the health of children in Europe by facilitating the development and availability of medicines for children aged 0–17 years. In 2010, around 21% of Europeans were children (>100 million people). However, studies carried out before the Regulation was adopted showed that >50% of the drugs used had not been tested in child populations [61].

Compounding represented 80% of all prescriptions until 1950s. Today, >90% of medicinal products are of industrial origin. Unfortunately, the legislation has not always evolved to stay in line with the public protection requirements [49]. In the wake of the New England Compounding Center scandal [62], the FDA put together a draft guideline to avoid compounding being used to create copies of approved products [49]. In Europe as well, compounding should be restricted to its original purpose, which is to provide a named-patient base solution to an individual patient need. As such, the HTAs should not endorse the use of compounded drugs manufactured on a wide scale, off-label, through granting them optimal – and one could argue preferential – reimbursement conditions in the presence of on-label alternatives.

A ‘Compounding Act’ in line with the spirit of the above-mentioned Pediatric Regulation would enable pharmaceutical companies to bring to the market standardized, tested, on-label drugs for the welfare of European patients. Another benefit would be to enable wider, more equalitarian access of drugs to the patient population. Indeed, a major drawback of compounded drugs is their access [36], as they are restricted to patients treated in a hospital setting.

Both HAS and NICE acknowledged the unmet need in the severe forms of DED. It is important from a societal perspective that resource allocation supports the ageing population living longer with a better quality of life and with functional independence. Maintaining vision is critical to human well-being, and interventions meant at breaking the circle of inflammation in DED can have a long-term benefit and positive impact on society as a whole – for patients and caregivers alike.
